# Shifting the Blame: How Narrative Framing, Coercive Strategies, and Rape Myth Acceptance Distort Perceptions of Sexual Assault and Fuel Victim Blame

**DOI:** 10.3390/bs16061039

**Published:** 2026-06-22

**Authors:** Pantxika Victoire Morlat, Maria Limniou, Isobel Phelps, Laurence Alison

**Affiliations:** Department of Psychology, University of Liverpool, Liverpool L69 7ZA, UK

**Keywords:** victim blaming, rape myth acceptance, perception, sexual assault, coercion, narrative, framing, physical force, alcohol

## Abstract

Previous research has shown that both victim intoxication and narrative framing can influence the levels of victim blame. However, far less attention has been paid to how coercive strategy and narrative framing may interact to shape victim-blaming judgements and perceptions of sexual assault. The present study addresses this gap by examining how combinations of coercive strategies (physical force versus alcohol facilitated), narrative framing (active versus passive), and rape myth acceptance (RMA) influence victim blame and the recognition of sexual assault. Participant gender and age were also assessed in relation to RMA and victim-blaming attitudes. A total of 202 participants aged 18–63 (78.7% of women, 21.3% of men, *M_Age_* = 28.93, *SD* = 14.36) completed an online survey evaluating vignettes depicting a male perpetrator sexually assaulting a female victim. Age significantly predicted victim blaming, with older participants assigning greater blame to the victim. Gender predicted both RMA and victim blame, with men reporting higher RMA and greater victim blame than women. Active framing in both the physical force and alcohol-use conditions reduced participants’ recognition of the incident as sexual assault. Participants with lower RMA consistently reported lower victim blame across conditions, and were more likely to identify the incident as sexual assault in the physical force condition. These findings highlight the influence of coercive strategies and the importance of victim-centred language in policing, legal, and media contexts, where narrative framing can meaningfully shape the recognition of sexual assault.

## 1. Introduction

“If thought corrupts language, language can also corrupt thought.”

(Orwell, 1946).

Research has demonstrated that coercive strategies used by perpetrators influence the levels of victim blame and perceptions of sexual assault (Dyar et al., 2021; Rogers et al., 2010; Romero-Sánchez et al., 2012; Russell et al., 2011). Yet, the mechanisms through which their influences operate remain incompletely understood. One underexplored dimension is narrative framing, which refers to the linguistic structure used to describe an event. In passive framing, the victim is positioned as being acted upon, whereas active framing depicts the victim as somehow participating in the action. These subtle linguistic differences shift perceived responsibility, agency, and intent. Understanding how coercive strategies and narrative framing jointly shape perceptions is essential, as both factors frequently appear in police reports, legal arguments, and media accounts of sexual assault.

Physical force and alcohol represent two common strategies but may operate through meaningfully different mechanisms and elicit distinct cognitive and moral evaluations from observers. Crucially, each strategy disrupts consent in different ways, and it is precisely at this intersection that rape myth acceptance (RMA) exerts its distorting influence. When a victim’s experience involves alcohol rather than physical force, widely held cultural scripts around personal responsibility and risky behaviour activate forms of blame that are not equally triggered by force-based assault (Romero-Sánchez et al., 2012). As Taylor (2020) identifies, victim blame embedded in rape myths takes three forms: situational blame (e.g., being in a deserted area), behavioural blame (e.g., drinking alcohol, or dressing provocatively), and characterological blame (e.g., being perceived as naïve or promiscuous). Indeed, it has been found that alcohol-facilitated assault is particularly vulnerable to behavioural blame attribution, in a way that physically coerced assault typically is not (Romero-Sánchez et al., 2012).

Thus, the present study addresses this gap by examining how combinations of coercive strategies (physical force versus alcohol), narrative framing (active versus passive), and RMA influence victim blame and sexual assault perception. Gender and age were also examined as predictors of these attitudes. By clarifying these perceptual mechanisms, the study aims to help inform educational and policy interventions, particularly within law enforcement and media communication, to reduce victim-blaming attitudes and support the more accurate recognition of sexual assault.

### 1.1. Consent for Sexual Contact

While consent is commonly defined as a person’s free, informed, and voluntary agreement to sexual activity, feminist scholarship highlights that the meaning is deeply embedded in political, economic, and social structures based on gendered expectations (Harris, 2018). The *no means no* model, emerging in the 1980s–1990s, redefined consent as the obligation to respect refusals; however, it was criticised for failing to address situations where a verbal ‘no’ is absent, most relevantly, intoxication or compliance under pressure. In response, the *yes means yes* or affirmative consent requires a clear, enthusiastic, and voluntary agreement, shifting responsibility onto sexual partners to ensure that consent is actively communicated rather than assumed (Popova, 2019). More recent sex-critical perspectives argue that consent cannot be understood solely at the individual level; instead, it must be viewed as relational and shaped by socio-political structures, power dynamics, and cultural scripts (Beres, 2014; Cahill, 2016; Fischel, 2016; Porter, 2024; Healey, 2022). In this view, consent is meaningful only when individuals have genuine freedom to say both *yes* and *no*. Phipps’s (2026) work further situates sexual consent within a colonial frame, recognising that some groups are purposely made more vulnerable and less protected, producing structural barriers that restrict autonomous sexual choices.

Nonconsensual sex is defined as ‘any sexual exchange where free or unfettered sexual consent is compromised’ (Farvid & Saing, 2021, p. 1379). This includes forced intercourse but also situations in which victims do not actively resist yet do not provide genuine consent. Perpetrators may use sexual coercion, including psychological or verbal pressure, to obtain sexual compliance (Brewer & Forrest-Redfern, 2022). In this study, the authors use the term ‘coercion’ to refer to assault scenarios involving either physical force or alcohol-facilitated incapacitation. Victims may respond with voluntary sexual compliance, agreeing to unwanted sex due to fear of consequences or as a survival strategy (Brewer & Forrest-Redfern, 2022; Farvid & Saing, 2021; Mann, 2023). It is in these ambiguous scenarios, where the absence of a verbal ‘no’ may be misread as implicit consent, that RMA plays an important consequential role. By normalising coercive or non-consensual scenarios and shifting responsibility onto victims, RMA distorts the evaluation of whether consent was genuinely present (Burt, 1980). Whilst the two coercive strategies examined in the present study, physical force and alcohol-facilitated incapacitation, both impair consent capacity, they tend to elicit different evaluations that are informed by existing attitudes and beliefs (Romero-Sánchez et al., 2012). Force-based coercion more closely resembles the ‘classic rape’ stereotype and thus tends to be judged as clearer-cut. By comparison, alcohol-facilitated coercion activates behavioural blame scripts that may question victim agency and responsibility, reducing the recognition of the absence of consent (Lussos & Fernandez, 2018; Ham et al., 2019). Narrative framing can further distort evaluations by presenting the victim as an active participant rather than being acted upon, which can wrongly suggest agency, thereby masking the absence of consent. These interacting influences, consent ambiguity, RMA, and linguistic framing, form the central theoretical motivation for the present study.

### 1.2. Victim-Blaming Attitudes

Victim blame, originally defined by Ryan (1971), refers to shifting responsibility from the perpetrator onto the victim. A substantial body of research shows that men tend to blame victims more than women (Grubb & Harrower, 2008; Martini & De Piccoli, 2020; Snow et al., 2023), a pattern aligned with feminist analyses arguing that misogynistic values and patriarchal norms position women as responsible for the victimisation they experience (Brownmiller, 2013). Age-related patterns are less consistent. Rankin (2000) found that older participants (60–79 years) were more likely to blame victims than younger adults (20–39 years), potentially reflecting more conservative ideology orientations (Adams-Price et al., 2004). In contrast, Chapin and Coleman (2017) found that 27.0% of middle and high school students believed that ‘victims brought it on themselves’ (p. 438) and observed a decrease in victim-blaming attitudes with age. However, because that study focused on bullying rather than sexual assault, these developmental trends may not be directly transferable to sexual assault contexts.

Overall, victim blame appears to emerge from a complex interaction of sexist and prejudicial beliefs that shape societal responses to victims’ own self-attributions (Taylor, 2020). MacLeod (2016) further explains how such expectations surface in police interviews, where victims are implicitly judged against standards of resistance or escape. For example, when a victim described being too intoxicated to move, the interviewer responded with questions such as the following: interviewee—‘I *couldn’t* move, I was that drunk, I *couldn’t* lift my body up I just felt’; response of the interviewer—‘*Could you* have sat up at all? […] *Could you* have got out of bed’ (p. 106). These expectations can undermine the victim’s experience and inadvertently generate empathy for the perpetrator. Narratives, whether institutional, cultural, or interpersonal, also shape how individuals interpret sexual assault scenarios and may guide them toward victim-blaming conclusions. Understanding how such narrative cues influence perceptions formed a central motivation for the present study.

### 1.3. Perceptions of Sexual Assault and RMA

Williams (1984) argues that the ‘classic rape’ stereotype involves a stranger perpetrator applying extreme physical force resulting in visible injury. In reality, many assaults involve verbal coercion, manipulation, or intoxicants, and victims may experience ‘tonic immobility’, a temporary involuntary paralysis during trauma (Mann, 2023). When victims’ experiences do not align with widely held rape beliefs, they may be reluctant to report the assault due to the anticipated blame or disbelief (Stewart et al., 2024). A total of 60.4% of victims do not label their experiences as rape despite meeting the legal criteria (Wilson & Miller, 2016), underlining the influence of rape myths, culturally embedded beliefs that justify or deny sexual aggression (Lonsway & Fitzgerald, 1994; Williams, 1984). RMA is strongly associated with hostile attitudes towards women (Suarez & Gadalla, 2010), reflecting broader sexist ideologies. The alcohol condition is particularly susceptible to behavioural blame because cultural narratives often interpret alcohol consumption as a form of risk-taking, leading observers to view the victim as partly responsible and less deserving of sympathy (Grubb & Turner, 2012; Romero-Sánchez et al., 2012). This logic is reinforced by evidence that perpetrators are often judged less harshly when intoxicated, whereas victims who had been drinking face increased blame (Cornelius et al., 2024; Martin & Monds, 2024).

Christie’s (1986) concept of the ‘ideal victim’ further illustrates how societal expectations shape perceptions of sexual assault. Victims who do not fit the stereotype of a defenceless, unsuspecting woman attacked by a stranger may be judged as less credible or more responsible for assault. This framework also fails to address the intersection between race, class, and victimisation. In England and Wales, Mixed and ‘Other’ ethnic groups experience higher victimisation than Whites (UK Government, 2024); yet, racialised policing and the framing of assaults as hate crimes rather than sexual crimes can obscure victims’ experiences and shift the focus to perpetrators’ motives. Similarly, migrant populations have often been dismissed from generalist frameworks of victimology, lacking both the identification of their vulnerabilities (Padovese & Rossoni, 2026) and their representations in sexual violence research (De Schrijver et al., 2018). Sex workers are likewise systematically excluded from victim idealisation, positioned as sexually available and thereby always consenting, despite empirical evidence confirming that they face high levels of blame for sexual violence (Sprankle et al., 2018; Sweeney & Sweeney Batard, 2024).

This aligns with Lips’s (2018) analysis of sexism as a system that normalises male dominance and aggression while positioning women as compliant and passive. Gendered expectations around sexual behaviour further reinforce these myths. Long-standing cultural narratives suggest that men desire sex more than women and are therefore expected to initiate physical intimacy. Media portrayals of heterosexual courtship often depict women’s initial refusal as part of a normative script, encouraging men to persist until boundaries are overcome (Trottier et al., 2025). Such narratives can legitimise coercive behaviour and shape public interpretations of sexual encounters. Recent Western-based research confirms the persistence of RMA. In France, Salmona (2019) found that 57.0% of respondents believed men struggle to control their sexual urges and 42.0% excused rapists if the victims behaved provocatively. In Quebec, Baril et al. (2025) reported a high agreement with statements minimising male responsibility, including that ‘sometimes they get sexually carried away’ (82.7%) and ‘Women […] sometimes gave men false hopes and later regretted it’ (81.3%). Perceptions of severity also vary by act: when genital penetration is absent, observers often minimise the harm (Mortimer et al., 2019), reinforcing a hierarchy of sexual violence. These patterns are directly relevant to the present study’s examination of how individual differences in RMA shape victim blame and the perceptions of sexual assault under the two coercive conditions.

### 1.4. Sexual Assault Narratives and Linguistic Framing

The public understanding of sexual assault is shaped by entrenched narrative templates. The ‘classic rape’ narrative, involving physical harm and overt force, continues to dominate lay beliefs, reinforcing the assumption that visible injury is a defining feature of assault (O’Neil & Morgan, 2010). Other factors, including victim attire, alcohol or drug use, and perceived character, further contribute to heightened victim-blaming (Krahé et al., 2008; Rinehart et al., 2023). Population-level data in England and Wales illustrate how these factors intersect with real cases: 39.0% of rape victims reported that perpetrators had consumed alcohol and 34.0% reported being under the influence themselves (Office for National Statistics [ONS], 2021). Among victims assaulted by strangers, 64.0% stated that the perpetrator had consumed alcohol and 54.0% reported the use of physical force (Office for National Statistics [ONS], 2021). Relyea and Ullman (2014) highlight that many women are reluctant to disclose voluntary alcohol consumption prior to the assault due to the anticipated negative reactions that can intensify stigma and self-blame. These dynamics reflect the broader tendency of behavioural blame attribution, as identified by Taylor (2020), to locate responsibility in the victim’s prior actions rather than the perpetrator’s conduct. The present study builds directly on Romero-Sánchez et al. (2012), who found that, when the male perpetrators used force rather than providing alcohol, Spanish participants attributed less blame to female victims and were more likely to classify the incident as sexual assault, a pattern consistent with the differential activation of behavioural blame scripts.

Narratives also influence blame through linguistic structure. Media portrayals often use passive or agentless constructions that minimise perpetrator responsibility and shift attention away from the violence (Gravelin et al., 2024). Taylor (2020) argues that institutional terminology such as ‘Violence Against Women and Girls’ (VAWG) inadvertently centres women as the locus of the problem, obscuring male perpetration. Similarly, Ehrlich (2003) notes that the phrase ‘allegations of sexual assault’ removes the responsible agent, reframing the situation as a legal or reputational threat to the accused rather than an experience of violence by the victim. Such phrasing can subtly frame the situation as something happening to the accused, a reputational or legal threat, while the victim’s experience becomes linguistically marginalised (Burrell, 2016). Cultivation-based research further shows that media consumption shapes beliefs about gender and sexual violence, with general television viewing being associated with an increased acceptance of rape-related attitudes (Fox & Potocki, 2016), suggesting that repeated exposure to distorted narratives normalises harmful assumptions.

### 1.5. Overview and Hypotheses

The preceding account identifies a web of mutually reinforcing influences: consent ambiguity arising from different coercive strategies, RMA beliefs that shape interpretations of that ambiguity in ways that disadvantage victims, and narrative framing that can increase perceived victim agency or reduce perceived perpetrator responsibility. Gender and age provide a further lens. Rape myths are deeply rooted in gender stereotypes that blame women for victimisation and normalise sexual aggression in men (Suarez & Gadalla, 2010), and older adults are often more likely to endorse conservative social attitudes and traditional gender norms (Adams-Price et al., 2004; Rankin, 2000).

Sexual assault remains a major public health and criminal justice concern. In England and Wales, an estimated 900,000 adults have experienced sexual assault since the age of 16 (739,000 females and 162,000 males; Office for National Statistics [ONS], 2025); yet, only 16.0% of incidents are reported to police (Office for National Statistics [ONS], 2021). Victims commonly cite the anticipated embarrassment, fear of not being believed, and perceptions that the police cannot help as reasons for non-disclosure (Office for National Statistics [ONS], 2021), responses reflecting the victim-blaming attitudes and RMA that are the focus of this study. Extending Romero-Sánchez et al. (2012), whose sample consisted exclusively of college students aged 18–28, the present study examined RMA, victim-blaming attitudes, and perceptions of sexual assault across a broader adult sample using vignettes varying by narrative framing (active versus passive) and coercive strategy (physical force versus alcohol).

**Figure 1:**
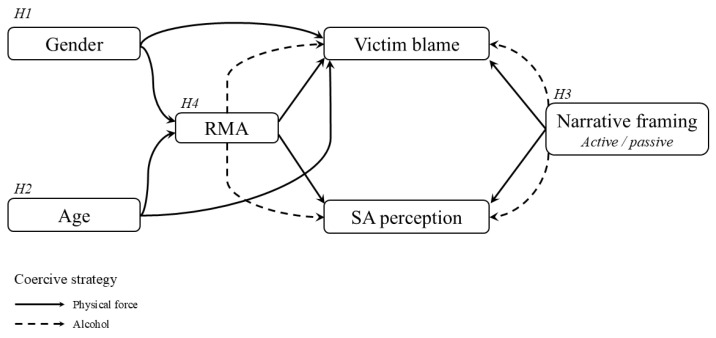
Conceptual map of hypothesised relationships. Note: RMA = rape myth acceptance; SA = sexual assault.

The present study tested four hypotheses, which are illustrated in Figure 1:

**H1.**  *Men report higher RMA and greater victim blame than women.*

**H2.**  *Older participants report higher RMA and greater victim blame than younger participants.*

**H3.**  *Victim blame and perceptions of the incident as sexual assault differ between the physical force and alcohol conditions, depending on whether the narrative framing is active or passive.*

**H4.**  *Participants with higher RMA report greater victim blame and are less likely to classify the incident as sexual assault than participants with lower RMA, with these effects varying according to whether the coercive strategy involves alcohol or physical force.*

## 2. Materials and Methods

### 2.1. Participants

A total of 202 participants took part in the study (78.7% of women, 21.3% of men) aged 18–63 years (*M_Age_* = 28.93, *SD* = 14.36). Participants were recruited through volunteer convenience sampling via social media advertisements, campus posters, and the University’s internal participation point scheme. Given this recruitment approach, the sample is unlikely to be representative of the broader population, and findings should be interpreted accordingly. In particular, volunteer samples focused on a university-affiliated platform may disproportionately include individuals with higher educational attainment and, potentially, stronger pre-existing engagement with issues of gender and sexual violence, either inflating or attenuating RMA scores relative to a general adult population.

The eligibility criteria required participants to be adults (≥18 years old) and fluent readers of English. The study was designed to examine differences between men and women. The authors acknowledge that this is a limitation as it forecloses examination of how non-binary and gender non-conforming individuals perceive sexual violence, a question of both theoretical and social importance that future research should address.

An a priori power analysis was conducted using G*Power 3.1.9.7 to determine the required sample size for the planned analyses (Faul et al., 2007). For multiple regression, assuming a small-to-medium effect size (*f*^2^ = 0.10–0.15), α = 0.05, and power of 0.80, the required sample size ranged from approximately *N* = 74 to *N* = 110. For group comparisons, a minimum of approximately *N* = 176 was required to detect medium effects (*d* = 0.50). The final sample (*N* = 202) exceeded the sample requirement.

### 2.2. Materials

The study was administered online using the web-secure Qualtrics survey platform. Participants first completed demographic questions (age and gender) before being randomly allocated to one of two vignette sequences by selecting either 1 or 2. Crucially, participants selected this number before any study content was presented to them; they were therefore naïve to which vignette combination they would receive at the point of allocation. This allocation procedure was designed to minimise demand characteristics by preventing participants from self-selecting into a condition that matched their prior attitudes.

#### 2.2.1. Experimental Vignettes

The hypothetical sexual assault vignettes were adapted from Romero-Sánchez et al. (2012) and translated from Spanish to English, with names Anglicised (Juan to John and Alicia to Alice). Each vignette described two strangers meeting at a bar, interacting socially and then encountering a situation in which John attempts to escalate physical intimacy despite Alice’s prior refusal. Two experimental manipulations are embedded for coercive strategy (physical force, with John ‘grabs her strongly’; or alcohol, in which John ‘decided to buy her shots of whisky so he can go further with her’), and narrative framing (passive: ‘John kisses her and touches her sexually’, and active: ‘they end up kissing and touching each other sexually’). The active framing subtly implies greater agency on Alice’s part through the use of plural pronouns (‘they’ or ‘each other’), despite the coercive context being identical across versions. The behaviours described meet the legal definition of sexual assault under Section 3 of the Sexual Offences Act 2003 (Crown Prosecution Service, 2026^1^).

Each participant read two vignettes, one with active framing and one passive framing, crossing with physical force or alcohol as the coercive strategy. The number (1 or 2) chosen at the outset determined which combination each participant received. For example, a participant who first reads an active/alcohol vignette subsequently reads a passive/physical force vignette (see Appendix A). This within-subjects exposure to both narrative frames, while keeping coercive strategy between participants, was designed to ensure sensitivity to framing effects while avoiding the transparency and fatigue associated with presenting all four conditions to a single individual.

#### 2.2.2. Sexual Assault Perception

Participants rated whether the outcome of each vignette constituted sexual assault using a single item from Romero-Sánchez et al. (2012): “The outcome of this story is a sexual assault”. Responses were on a 7-point Likert scale (1 = Completely disagree, 7 = Completely agree). Higher scores indicated stronger perception of the event as a sexual assault.

#### 2.2.3. Victim-Blaming Attitudes

Victim blame was assessed using six items from Romero-Sánchez et al. (2012), including the following: “Alice could have prevented what happened at the end of this story” and “Alice could have behaved differently to change the outcome of this story”. Responses were on a 7-point Likert scale (1 = Strongly disagree, 7 = Strongly agree). Higher scores indicated greater victim blame. Internal consistency in the present study was adequate (*α* = 0.72).

#### 2.2.4. RMA

RMA was measured using the shortened 16-item version of the Acceptance of Modern Myths about Sexual Aggression scale (AMMSA; Gerger et al., 2007), as used by Romero-Sánchez et al. (2012). Example items include the following: “Interpreting harmless gestures as ‘sexual harassment’ is a popular weapon in the battle of the sexes” and “Many women tend to misinterpret a well-meant gesture as a ‘sexual assault’”. Responses were on a 7-point Likert scale (1 = Completely disagree, 7 = Completely agree). Higher scores indicated stronger endorsement of rape myths. Reliability in the present study was very good (*α* = 0.88).

### 2.3. Procedure

Participants accessed the study online and first read an information sheet before providing informed consent. They then completed demographic questions and selected a number (1 or 2) to determine their vignette sequence. After reading each vignette, they completed measures of perception of sexual assault and victim-blaming attitudes. After completing both vignettes, participants completed the RMA scale. Upon completion, participants were debriefed and provided with support resources (e.g., Rape Crisis England & Wales), in case they experienced distress related to the study content.

### 2.4. Design

Four hypotheses were tested using a combination of multiple linear regressions and Welch one-way analyses of variance (ANOVAs).

For H1 and H2, considering the roles of gender and age in predicting RMA and victim-blaming attitudes, separate multiple linear regression analyses were specified, with gender and age being entered simultaneously as independent variables (IVs). Multiple regression was selected over simple bivariate tests because it allows both predictors to be examined while statistically controlling for the other, providing a more accurate estimate of each variable’s independent contribution. For H1, the dependent variable (DV) was RMA score, and, for H2, the DV was victim-blaming score.

For H3, examining whether victim blame and sexual assault perception differ between narrative framing (active versus passive) across the two coercive contexts, Welch one-way ANOVAs were conducted separately for the physical force and alcohol conditions. Welch’s correction was applied in preference to standard ANOVA because of its robustness to violations of the homogeneity of variance assumption. The IV was narrative framing condition (active versus passive), and the DVs were victim-blaming scores and sexual assault perception scores.

For H4, measuring whether RMA level predicts victim blame and sexual assault perception, participants were median-split into high and low RMA groups, and Welch one-way ANOVAs were again conducted separately for each coercive strategy condition. The IV was RMA group (high versus low), and the DVs were victim-blaming scores and sexual assault perception scores. The use of median splits to create RMA groups was adopted to maintain comparability with Romero-Sánchez et al.’s (2012) study.

## 3. Results

The data were analysed using IBM Statistical Package for the Social Sciences (SPSS) Statistics version 29. Less than 5% of cases contained missing data and were excluded using listwise deletion, and no systematic patterns of missingness were identified. The significance threshold for H1 and H2 was set at *p* < 0.05. Because H3 and H4 involved multiple related comparisons, a Bonferroni-adjusted alpha level of *p* < 0.0125 was applied to control for the familywise Type I error (Abdi, 2007).

### 3.1. H1 and H2: Gender and Age as Predictors of RMA and Victim Blame

A multiple regression analysis was conducted to examine whether gender and age predicted RMA. All assumptions of multiple regression were assessed and met, including linearity, homoscedasticity, the independence of errors, the normality of residuals, and the absence of multicollinearity. Table 1 presents the full results for both models, including unstandardised coefficients (*B*), standard errors, standardised coefficients (β), and the 95% confidence intervals for each predictor. Model-level fit statistics are reported in the table footer.

#### 3.1.1. RMA

**Figure 2:**
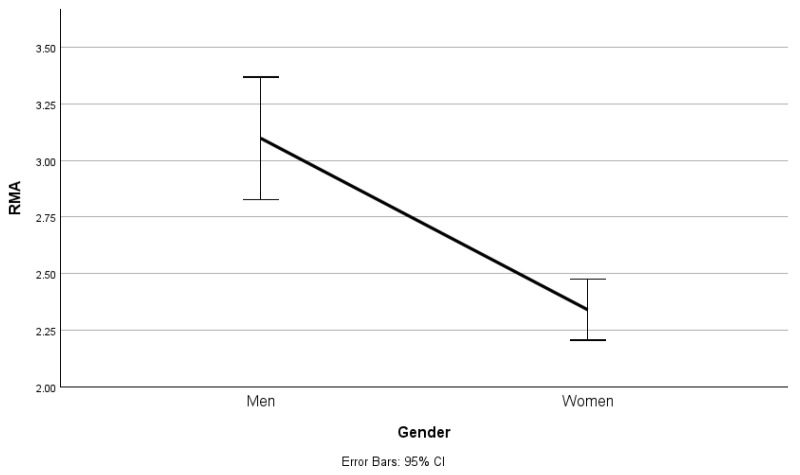
Gender differences in RMA scores. Note: RMA = rape myth acceptance.

The overall model was significant, with adjusted *R*^2^ = 0.11, *F*(2, 198) = 13.48, *p* < 0.001, and *f*^2^ = 0.14, indicating that gender and age together accounted for 11% of the variance in RMA scores, a small-to-medium effect by conventional benchmarks (Cohen, 1988). Gender significantly predicted RMA, with *B* = −0.78, β = −0.35, *SE* = 0.15, *t*(198) = −5.19, *p* < 0.001, and 95% CI [−1.08, −0.48]. Men reported higher RMA scores (*M* = 3.10, *SD* = 0.88) than women (*M* = 2.34, *SD* = 0.86), with a one-unit increase in gender coding (1 = man, 2 = woman) associated with a 0.78-point decrease in RMA scores (see Figure 2). In contrast, age was not a significant predictor of RMA, with *B* = −0.00, β = −0.06, *SE* = 0.00, *t*(198) = −0.88, and *p* = 0.383.

Overall, H1 was partially supported, as gender significantly predicted RMA whereas age did not.

#### 3.1.2. Victim Blame

The regression model for victim blame was significant and explained a larger share of the variance than the RMA model, with adjusted *R*^2^ = 0.18, *F*(2, 199) = 22.78, *p* < 0.001, and *f*^2^ = 0.23, indicating a medium effect size and explaining 18% of the variance in victim-blaming scores.

**Figure 3:**
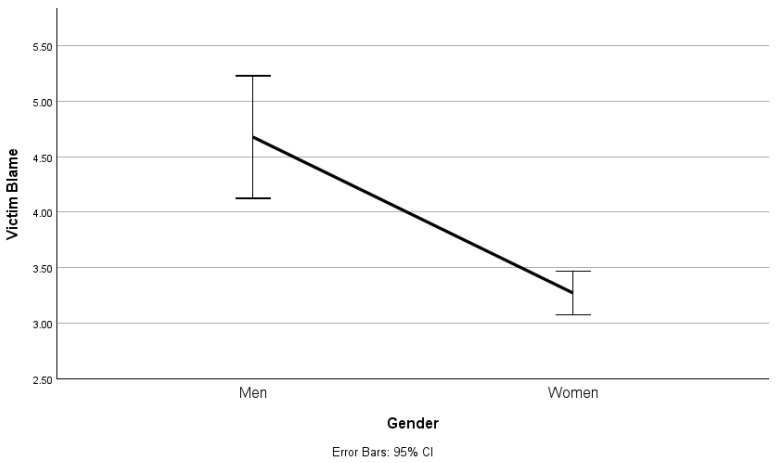
Gender differences in victim-blaming scores.

Gender significantly predicted victim blame, with *B* = −1.30, β = −0.36, *SE* = 0.24, *t*(199) = −5.49, *p* < 0.001, and 95% CI [−1.77, −0.83]. Men reported higher victim-blaming attitudes (*M* = 4.68, *SD* = 1.80) than women (*M* = 3.27, *SD* = 1.26; see Figure 3).

**Figure 4:**
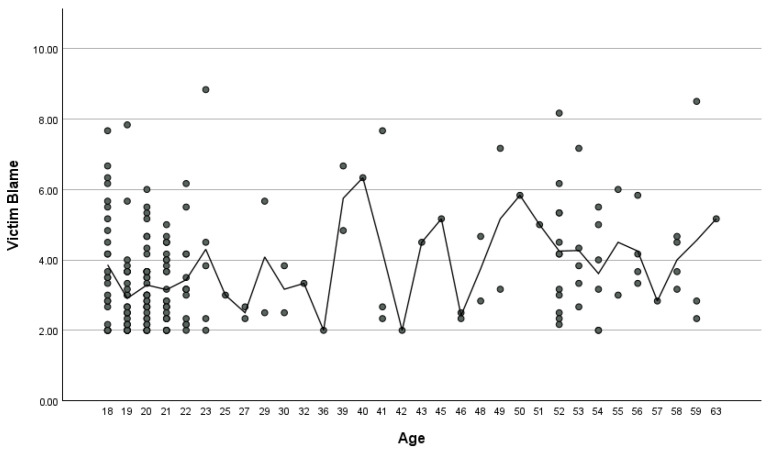
Association between age and victim-blaming scores.

Age also significantly predicted victim blame, with *B* = 0.02, β = 0.20, *SE* = 0.01, *t*(199) = 3.05, *p* = 0.003, and 95% CI [0.01, 0.03], indicating that older participants were more likely to attribute blame to the victim (see Figure 4).

These findings support H2, demonstrating that both gender and age were significant predictors of victim-blaming attitudes (see Table 1 for a summary of H1 and H2).

### 3.2. H3: Narrative Framing, Victim Blame, and Perceptions of Sexual Assault

A series of Welch one-way ANOVAs examined whether narrative framing (active versus passive) influenced victim blame and sexual assault perception across the two coercive strategies (physical force and alcohol). Welch’s correction was applied due to violations of the homogeneity of the variance.

#### 3.2.1. Victim Blame

In the physical force condition, participants exposed to the active narrative reported slightly higher victim-blaming attitudes (*M* = 1.87, *SD* = 0.88) than those in the passive narrative condition (*M* = 1.64, *SD* = 0.72). This effect was statistically significant prior to correction, with Welch’s *F*(1, 182) = 4.35, *p* = 0.039, *ω*^2^ = 0.01, and 95% CI [−0.01, 0.07], but did not meet the Bonferroni-adjusted threshold of *p* < 0.0125.

Similarly, in the alcohol condition, victim blame was descriptively higher in the active condition (*M* = 1.92, *SD* = 1.01) than in the passive condition (*M* = 1.71, *SD* = 0.77), although this difference was not statistically significant, with Welch’s *F*(1, 127.06) = 2.38, and *p* = 0.125.

#### 3.2.2. Perceptions of Sexual Assault

Narrative framing had a stronger effect on participants’ perceptions of sexual assault. In the physical force condition, participants perceived the vignette as more clearly depicting sexual assault when the narrative was written in the passive condition (*M* = 6.70, *SD* = 0.59) compared to the active condition (*M* = 5.60, *SD* = 1.27), with Welch’s *F*(1, 190.05) = 70.26, *p* < 0.001, *ω*^2^ = 0.20, and 95% CI [0.11, 0.29]. This large effect indicates that narrative framing accounted for 20% of the variance in perceptions of sexual assault.

A similar directional pattern emerged in the alcohol condition, where passive framing was associated with higher perceptions of sexual assault (*M* = 6.34, *SD* = 0.82) than active framing (*M* = 5.91, *SD* = 1.31), with Welch’s *F*(1, 111.13) = 6.74, *p* = 0.011, *ω*^2^ = 0.04, and 95% CI [−0.00, 0.10]. This effect, while statistically significant at the Bonferroni-adjusted threshold, was small: narrative framing explained only 4% of the variance in the alcohol condition, compared to 20% in the physical force condition. These findings partially support H3. A passive narrative framing consistently increased the recognition of sexual assault across both coercive contexts, particularly in the physical force condition. However, narrative framing did not significantly influence victim-blaming attitudes.

### 3.3. H4: RMA, Victim Blame and Perceptions of Sexual Assault

Welch one-way ANOVAs examined whether RMA (high versus low) predicted victim blame and sexual assault perception across the two coercive strategies.

#### 3.3.1. Victim Blame

In the physical force condition, participants with high RMA scores reported significantly greater victim blame (*M* = 2.18, *SD* = 0.94) than participants with low RMA scores (*M* = 1.40, *SD* = 0.47), with Welch’s *F*(1, 141.72) = 54.02, *p* < 0.001, *ω*^2^ = 0.21, and 95% CI [0.12, 0.31]. This large effect indicates that RMA accounted for 21% of the variance in victim-blame in the physical force condition, comparable in magnitude to the combined contribution of gender and age to victim-blaming attitudes in H2.

The same pattern was observed in the alcohol condition, where participants with high RMA scores again attributed more blame to the victim (*M* = 2.09, *SD* = 0.94) than those with low RMA scores (*M* = 1.50, *SD* = 0.69), with Welch’s *F*(1, 176.95) = 25.49, *p* < 0.001, *ω*^2^ = 0.11, and 95% CI [0.04, 0.2]. This medium effect shows that RMA, accounted for 11% of the variance in victim blame, strongly shaping victim-blaming attitudes across coercive contexts.

#### 3.3.2. Perceptions of Sexual Assault

In the physical force condition, low-RMA participants were more likely to recognise the vignette as sexual assault (*M* = 6.32, *SD* = 0.93) than high-RMA participants (*M* = 5.69, *SD* = 1.34), with Welch’s *F*(1, 171.87) = 14.64, *p* < 0.001, *ω*^2^ = 0.07, and 95% CI [0.01, 0.14]. RMA accounted for 7% of the variance in participants’ recognition of a force-based assault as sexual assault, highlighting that the influence of RMA persisted even when the coercive nature of the incident was made explicit through the use of physical force.

In contrast, no significant differences emerged in the alcohol condition, with Welch’s *F*(1, 197.45) = 1.64, and *p* = 0.202. Descriptively, low-RMA participants reported slightly higher assault recognition (*M* = 6.27, *SD* = 1.03) than high-RMA participants (*M* = 6.08, *SD* = 1.07), but this difference was not statistically meaningful.

**Figure 5:**
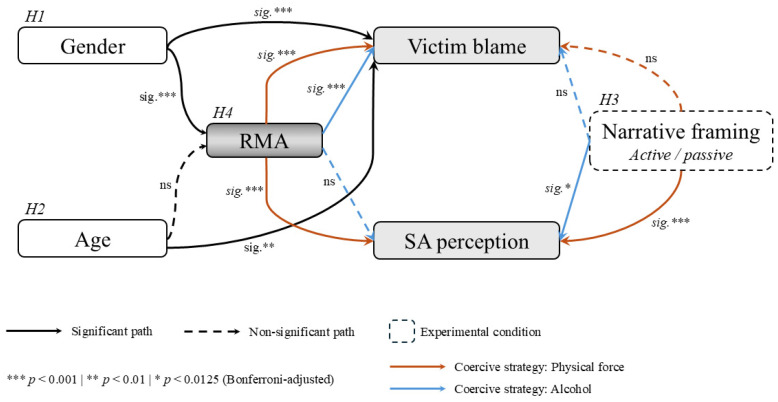
Conceptual map of tested relationships. Note: RMA = rape myth acceptance; SA = sexual assault.

Overall, H4 received partial support. Higher RMA consistently increased victim-blaming attitudes across both coercive contexts and reduced the recognition of sexual assault in the physical force condition, although this effect did not extend to the alcohol condition. Figure 5 presents an overview of the findings, illustrating significant and non-significant effects across experimental conditions.

## 4. Discussion

The present study examined how combinations of coercive strategies (physical force versus alcohol), narrative framing (active versus passive), and RMA influence victim blame and sexual assault perception, with gender and age as additional predictors. Using experimentally manipulated vignettes depicting non-consensual sexual contact, the study explored how subtle linguistic cues and coercive contexts shape judgements of victimisation and perpetration. The findings demonstrated that narrative framing and coercive strategies meaningfully alter how sexual assault scenarios are perceived, that RMA shapes both victim blame and assault recognition, and that these effects interact with gender and age.

### 4.1. Gender, Age, RMA, and Victim Blame

Consistent with a substantial body of literature, men reported higher RMA and greater victim-blaming attitudes than women (Grubb & Harrower, 2008; Suarez & Gadalla, 2010; Strömwall et al., 2013; Martini & De Piccoli, 2020). These findings align with the Defensive Attribution Theory (DAT; Shaver, 1970), which proposes that individuals distance themselves from victim with whom they share less perceived similarity. Because most rape victims are women, men may perceive lower personal relevance and therefore attribute more responsibility to the victim (van der Bruggen & Grubb, 2014). The System Justification Theory (Łyś et al., 2021) offers a complementary explanation, highlighting how patriarchal structures shape interpretations of sexual violence in ways that naturalise male dominance and position women as responsible for managing sexual risk. Cultural narratives that sexualise women or question their credibility (DiBennardo, 2018), and media practices that obscure structural inequalities (Kitzinger, 2005) further reinforce these gendered patterns. These frameworks suggest that gender differences in RMA and victim blame are not merely psychological but are reproduced through broader social systems, a point developed further in Section 4.3 below.

Age was positively associated with victim-blaming attitudes, echoing findings that older adults may endorse more conservative gender norms (Adams-Price et al., 2004). However, age did not significantly predict RMA. Victim blame, by contrast, may be more directly responsive to the conservative gender-role expectations associated with older age, expectations that position women as responsible for preventing sexual victimisation. Notably, younger participants may simultaneously reject overt rape myths while still engaging in modern sexist reasoning. Research indicates that beliefs minimising gender inequality persist among younger populations and may arise from the perceived competition between men and women (Henley et al., 1995; Off et al., 2022), particularly in contexts shaped by discourse such as the #MeToo movement (Chandra & Erlingsdóttir, 2021). Although victim blame and RMA are closely related, the present findings suggest they may be differentially affected by demographic factors.

### 4.2. Narrative Framing, Coercive Strategy, Victim Blame, and Sexual Assault Perception

Narrative framing significantly influenced perceptions of sexual assault across both coercive conditions. When vignette used active framing (‘they end up kissing and touching each other sexually’), participants were less likely to recognise the encounter as sexual assault than when passive framing positioned John as the unambiguous agent (‘John ends up kissing her and touching her sexually’). This demonstrates that subtle linguistic cues implying shared agency can distort perceptions of coercion, even when the perpetrator’s behaviour is explicitly described as coercive. Active framing appears to lead readers to infer mutual participation, thereby reducing the perceived harm and assault recognition. These findings are consistent with evidence suggesting that even limited or ambiguous accounts of victim behaviour can affect perceptions of culpability and increase victim-blaming judgements (Romero-Sánchez et al., 2012).

The framing effect was significantly stronger in the physical force condition than in the alcohol condition, which has important theoretical implications. In the physical force scenario, which closely matches a stereotypical ‘classic rape’ template, small grammatical shifts (e.g., “John kisses her” versus “they end up kissing”) were sufficient to reduce assault recognition. This supports Gravelin et al. (2024), who argue that agentive language can diffuse responsibility across both parties, and aligns with Ehrlich’s (2003) view that linguistic choices can reframe violence as mutual interaction. In contrast, assault recognition was lower overall in the alcohol condition and less affected by framing, suggesting that intoxication already introduces ambiguity around consent and responsibility, limiting the additional impact of the grammatical structure. Based on the vignettes, this resonates back to Taylor’s (2020) typology of victim blaming, arguing that situational (i.e., being in a bar at night), behavioural (i.e., drinking alcohol), and characterological blame (i.e., being flirty) may fuel justifications for considering the victim as somewhat responsible. This finding is consistent with prior work showing that alcohol-facilitated assaults are judged more harshly toward victims (Grubb & Turner, 2012; Romero-Sánchez et al., 2012). This is likely to be caused by alcohol consumption being entangled with cultural stereotypes about women’s behaviour, sexual availability, and responsibility for risk (Leverick, 2020).

### 4.3. Institutional Language and Structural Reproduction of RMA

RMA was strongly associated with victim blame across coercive conditions, and with the reduced recognition of sexual assault in the physical force condition. Participants with higher RMA consistently attributed more blame to the victim and were less likely to classify the force-based scenario as assault. These findings align with extensive evidence that rape myths function as socio-cognitive schemas that minimise perpetrator responsibility, exaggerate victim culpability, and normalise coercive sexual behaviour (Bohner et al., 2009; Süssenbach et al., 2017). Individuals high in RMA may selectively attend to details that confirm pre-existing beliefs, such as perceived victim behaviour, prior relationship with the perpetrator, and consumption of alcohol, while minimising or reinterpreting coercive actions. This interpretive bias also operates linguistically, as individuals high in RMA often use passive constructions that distance perpetrators from their actions (Bohner, 2001), mirroring and potentially reinforcing the framing effects observed in this study.

Importantly, RMA influenced judgments in the physical force condition, even where the perpetrator’s coercive strategies was explicit. This reinforces the robustness of rape myths; individuals who endorse them may reinterpret even clear scenarios in ways that reduce the perceived harm or shift responsibility onto the victim. The findings highlight the need for interventions that challenge rape-supportive beliefs, particularly among groups with high RMA. Returning to Taylor’s (2020) taxonomy, this suggests that behavioural blame, particularly drinking alcohol, may operate at a level of cultural embeddedness that transcends individual differences in measured RMA. This finding has direct implications for jury selection and legal training, where the assumption that low-RMA individuals will be neutral evaluators of alcohol-facilitated assault may be unwarranted.

The Just World Belief Theory (JWB; Lerner, 1980) also contributes to these patterns, such as the belief that bad things happen only to those who make poor choices encouraging victim responsibility attribution as a means of maintaining a sense of fairness and predictability (Grubb & Harrower, 2008; Strömwall et al., 2013). For some participants, including women, victim-blaming may also operate as a psychological defence mechanism, as distancing oneself from the victim reduces the discomfort associated with recognising that women are disproportionately targeted by sexual violence. This can create an illusion of control by attributing the assault to the victim’s choices (Mavin et al., 2014). In some cases, victims may even be accused of deriving pleasure from the encounter (Ayala et al., 2018; McDaniel & Rodriguez, 2021), or labelled as ‘promiscuous’, consistent with slut-shaming practices (Setty, 2020). This dynamic reinforces contradictory expectations placed on women. On one hand, patriarchal norms embedded in RMA position men as dominant and women as passive; yet, women are simultaneously expected to physically resist sexual assault to be considered ‘legitimate’ victims. This creates an untenable standard. Victims must embody the stereotype of the fragile, overpowered woman while also being criticised for not fighting back. When women do resist, they may challenge gendered assumptions about male strength and female vulnerability, and, in extreme cases, such as lethal self-defence, may even be reframed as aggressors (LaFortune, 2025). It is also important to acknowledge that women themselves may endorse patriarchal or misogynistic beliefs (Mavin et al., 2014).

These individual-level explanations, however, are insufficient on their own. RMA and victim blame do not persist solely through individual cognition; they are institutionally reproduced through the language of police reports, judicial proceedings, and media accounts. Phipps’s (2026) analysis of sexual violence within racial capitalism provides a structural frame for understanding this persistence. On this account, the preference for passive and agentless constructions in institutional discourse, such as ‘it was alleged that sexual contact occurred’, and ‘the complainant’s account was inconsistent’, are not neutral stylistic choices but a product of political and economic systems in which women and girls, and particularly those from racialised and economically marginalised groups, are kept structurally vulnerable and positioned as available to male violence. The institutional language that minimises perpetrator agency is the same language that limits the support available to victims and narrows the criteria by which victimisation is officially recognised. Taylor’s (2020) point that terminology such as ‘Violence Against Women and Girls’ inadvertently centres women as the focus of the issue, rather than explicitly naming, for instance, ‘Male Sexual Violence Perpetration’, which exemplifies a manifestation of the structural tendency in how sexual violence is framed. Another example of this effect is Ehrlich’s (2003) observation that the phrase ‘allegations of sexual assault’ removes the responsible agent. Both reflect a system in which language choices represent and reproduce the unequal distribution of protection and vulnerability that Phipps (2026) describes. The present study’s finding that passive framing significantly increases assault recognition is therefore not only a psycholinguistic result, but also evidence of the real perceptual consequences of a discursive environment in which agentless and mutualising language becomes the institutional norm.

### 4.4. Theoretical Contribution

The findings contribute to theoretical models of sexual assault perception by highlighting the role of language as a mechanism through which rape myths operate. While the Defensive Attribution Theory and Just World Belief Theory explain why individuals distance themselves from victims (Pinciotti & Orcutt, 2020), the present study shows that narrative framing can amplify or attenuate these biases by altering the perception of sexual assault. The interaction between coercive strategy and narrative framing suggests that existing theories must account for the interplay between linguistic cues and contextual ambiguity (Bohner, 2001). Moreover, the strong influence of RMA across all outcomes reinforces its role as a socio-cognitive schema that shapes victim blaming and the interpretation of sexual assault, extending the prior work by demonstrating that RMA moderates the impact of narrative framing itself (Elmore et al., 2021; Jozkowski et al., 2019). It is also important to consider the choice of language as politicised and deliberate (Phipps, 2026). These findings heightened the need for theoretical models that integrate language, context, and belief systems to explain how judgments of sexual assault are formed.

### 4.5. Strengths and Limitations

The study has several methodological strengths. The mixed design allowed for a nuanced examination of narrative framing within participants while avoiding the fatigue associated with repeated exposure to coercive strategies (Bohner, 2001). The sample exceeded the power requirements for both regression and group-based analyses. Validated measures of RMA and victim-blaming attitudes ensured a strong psychometric reliability (PettyJohn et al., 2023; Romero-Sánchez et al., 2012). The agency manipulation through active versus passive framing represents a methodological contribution, demonstrating that subtle narrative choices measurably alter assault perception (Lussos & Fernandez, 2018). The inclusion of both physical force and alcohol-facilitated coercion increases ecological validity by reflecting the diversity of real-world assault scenarios (Ham et al., 2019).

However, the findings should be interpreted in light of several limitations. Each participant read two vignettes, which may have introduced fatigue or reduced attention (Lietz, 2010), and vignettes can allow for interpretive ambiguity, as readers may project their own assumptions onto the scenario (Agostini et al., 2024). The study was conducted in England using vignettes adapted from a Spanish study. Thus, cultural norms surrounding gender, consent, and sexual behaviour may vary across societies, particularly given the recent changes in Spanish law defining rape as ‘sex without consent’ (Burgen, 2022; Gregory, 2022). The findings may therefore not generalise to non-Western contexts or to countries with different legal frameworks.

Furthermore, gender was operationalised using a binary coding scheme. This is likely to simplify the complexity of gender identity and may obscure important differences in perceptions of victim blame and sexual assault. Future research should incorporate diverse gender configurations to examine whether victim and perpetrator sex/gender interact with narrative framing and coercive strategy.

Then, the Anglicisation of character names (Juan to John, and Alicia to Alice) carries implicit racialising effects that were not controlled for or measured. Names function as racial and ethnic cues that can activate stereotypical associations and influence blame attributions independently of the vignette content. Future research should manipulate the racial and ethnic coding of character names and examine their interaction with coercive strategy and narrative framing. This limitation neglects to address the experiences of racialised populations, who experience higher rates of victimisation alongside increased scepticism and racialised policing when seeking recognition as victims (Long, 2021; UK Government, 2024).

Further, the study did not address migration status as a dimension of vulnerability. Migrant populations face compounding barriers to disclosing assault and accessing support, including language barriers, a precarious legal status, and a distrust of institutions (De Schrijver et al., 2018; Padovese & Rossoni, 2026). Research examining how narrative framing and RMA shape responses to vignettes depicting migrant victims would be a meaningful extension.

Moreover, sex workers represent a population systematically excluded from the ‘ideal victim’ framework, yet are highly vulnerable to sexual violence and blame attribution (Sprankle et al., 2018; Sweeney & Sweeney Batard, 2024). Vignettes involving sex worker victims would allow an investigation of whether framing effects, RMA, and behavioural blame operate differently when the victims’ occupational status already triggers assumptions about sexual availability and consent, directly addressing Christie’s (1986) ideal victim concept. Vignettes including marginalised populations in sexual violence scenarios could more deeply assess the dynamics when a victim’s sexuality is already structurally stigmatised.

Finally, all vignettes depicted a heterosexual assault scenario involving a male perpetrator and female victim. While this reflects the most commonly represented pattern within sexual violence research, it limits the generalisation to LGBTQ+^2^ victimisation contexts, including same-sex, transgender, and non-binary experiences of sexual assault. Relatedly, the AMMSA scale was developed primarily within heterosexual assault frameworks and may not fully capture RMA to LGBTQ+ assault scenarios, for example, myths that minimise male-on-male assault or deny the possibility of rape within same-sex relationships. Future research should develop and validate RMA measures appropriate for LGBTQ+ experiences.

### 4.6. Practical Implications and Recommendations

The findings carry direct implications for law enforcement, legal practice, and media communication. A victim-centred approach to language, one that names the perpetrator as the agent and attributes responsibility clearly, may help counteract narratives that shift the blame onto the victims. Although law enforcement practitioners receive training in rapport-based interviewing, they still report challenges in eliciting accurate accounts and building trust with victims (Cassidy et al., 2020). Interview guidelines that emphasise non-judgemental communication and avoid the active framing of victim behaviour can reduce secondary victimisation during reporting and support more effective investigations (Kim et al., 2020; Morlat & Alison, 2026).

In legal settings, the present findings indicate that jurors may be influenced by narrative framing in ways that closely parallel the experimental effects observed here. This is particularly relevant in alcohol-facilitated cases, where jurors may rely more heavily on pre-existing schemas about consent and victim behaviour when situational ambiguity is high (Abbey et al., 2004; Abbey et al., 2014; Dinos et al., 2015; Leverick, 2020). Legal professionals should be alert to the narrative framing of evidence, particularly whether descriptions of the victim’s actions use active or mutualising language, and training programmes should address how linguistic choices shape perceptions of agency and consent. One of the most frequently cited reasons for not reporting sexual assault is a lack of confidence in the criminal justice system (Stewart et al., 2024); improvements in the linguistic care taken by investigators and prosecutors may, over time, increase victims’ willingness to come forward.

Media reporting shapes the public understanding of sexual assault, particularly in high-profile sexual assault cases (Kitzinger, 2005; Gravelin et al., 2024). Yusri et al. (2025) identifies three common forms victim-blaming narratives in media coverage, portraying the victim as attention-seeking, passive, or unaware. Such portrayals normalise victim blame and perpetuate misconceptions. Individuals high in RMA are more likely to use the passive constructions when describing rape (Bohner, 2001), and media reporting frequently mirrors these patterns. Guidelines for responsible reporting that avoid active or mutualising constructions and instead name perpetrator agency explicitly would both reflect and potentially reshape the public understanding of sexual assault.

Future research should examine how narrative framing interacts with additional contextual cues, including victim resistance, intoxication levels, and the perpetrator–victim relationship (Rinehart et al., 2023; Bošković et al., 2025). Expanding vignette designs to include diverse gender configurations, racialised character names, and marginalised victim identities, including sex workers and migrants, would test the generalisability of these findings and address the intersectional gaps identified above. Cross-cultural studies using adapted vignettes are also needed to explore how legal definitions and social norms moderate framing effects (Pincock et al., 2023; Wager et al., 2021). Experimental work with legal professionals and mock jurors could further examine how narrative framing affects decision-making in courtroom settings. Finally, intervention research should investigate whether targeted training in recognising and resisting active framing, for law enforcement, legal professionals, and journalists, can reduce victim-blaming bias in real-world judgements.

## 5. Conclusions

The present study addressed an important gap by examining how narrative framing and coercive strategies shape perceptions of sexual assault across gender and age. The findings demonstrate that perceived victim agency, manipulated through active versus passive narrative framing, significantly increased the perception of sexual assault, especially when physical force was used. These effects are closely intertwined with RMA, which emerged as strongly associated with both victim blame and a reduced recognition of sexual assault. Men reported higher RMA and greater victim-blaming attitudes than women, reflecting broader patterns of misogynistic and patriarchal beliefs that continue to shape interpretations of sexual violence. Victim-blaming attitudes were higher among older participants who may hold more conservative ideology. Overall, the evidence presented suggests that RMA permeates multiple layers of sexual assault. Addressing these myths through targeted training for law enforcement, legal and media professionals, is essential for reducing victim blame and preventing second victimisation during reporting and investigation. Improving the quality of interactions between victims and the criminal justice system may increase reporting rates, enhance trust, and support the more effective prosecution of offenders. Ultimately, dismantling rape myths is critical to shifting societal narratives toward the resounding truth that responsibility for sexual assault always lies with the perpetrator. 

## Figures and Tables

**Table 1:** Multiple regression analyses predicting RMA and victim blame from gender and age.

Predictor	B	*SE*	β	*t*	*p*	95% CI
Outcome Variable: RMA						
Gender	−0.78	0.15	−0.35	−5.19	<0.001	[−1.08, −0.48]
Age	−0.00	0.00	−0.06	−0.88	0.383	[−0.01, 0.01]
Outcome Variable: Victim Blame						
Gender	−1.30	0.24	−0.36	−5.49	<0.001	[−1.77, −0.83]
Age	0.02	0.01	0.20	3.05	0.003	[0.01, 0.03]

Note: RMA = rape myth acceptance.

## Data Availability

The raw data supporting the conclusions of this article will be made available by the authors upon request.

## Notes

1 Additional information on rape and sexual offences legislation in England and Wales is accessible on https://www.cps.gov.uk/legal-guidance/rape-and-sexual-offences-chapter-7-key-legislation-and-offences (accessed on 18 February 2026).

2 LGBTQ+ stands for lesbian, gay, bisexual, transgender, queer, and other gender and sexual minority groups.

## Abbreviations

The following abbreviations are used in this manuscript:

AMMSA	Acceptance of Modern Myths about Sexual Aggression
ANOVAs	Analysis of Variances
ONS	Office for National Statistics
RMA	Rape Myth Acceptance

## Appendix A. Experimental Vignettes Based on Romero-Sánchez et al. (2012)’s Study^3^

### Appendix A.1. Vignette One—Coercive Strategy: Alcohol Use; Narrative Framing: Active Voice

John is out with some friends in a bar in the city. He has been watching a girl for a while that he really likes but, until now, he has not decided to approach her. She is dancing with some friends and seems to be interested in him because she has glanced at him several times. John decides to approach her, and he introduces himself. She responds by saying that her name is Alice, and the two begin to talk as they laugh and dance. The night goes on and the two continue flirting and having a good time together. John is very attracted to Alice, and he would like to “take it to the next level” with her, but Alice turns him down several times. John, despite her constant rejection, decides to buy her shots of whisky so that he can go further with her. Finally, after Alice has had several whiskies, they end up kissing and touching each other sexually.

### Appendix A.2. Vignette Two—Coercive Strategy: Alcohol Use; Narrative Framing: Passive Voice

John is out with some friends in a bar in the city. He has been watching a girl for a while that he really likes but, until now, he has not decided to approach her. She is dancing with some friends and seems to be interested in him because she has glanced at him several times. John decides to approach her, and he introduces himself. She responds by saying that her name is Alice, and the two begin to talk as they laugh and dance. The night goes on and the two continue flirting and having a good time together. John is very attracted to Alice, and he would like to “take it to the next level” with her, but Alice turns him down several times. John, despite her constant rejection, decides to buy her shots of whisky so that he can go further with her. Finally, after Alice has had several whiskies, John ends up kissing her and touching her sexually.

### Appendix A.3. Vignette Three—Coercive Strategy: Physical Force; Narrative Framing: Active Voice

John is out with some friends in a bar in the city. He has been watching a girl for a while that he really likes but, until now, he has not decided to approach her. She is dancing with some friends and seems to be interested in him because she has glanced at him several times. John decides to approach her, and he introduces himself. She responds by saying that her name is Alice, and the two begin to talk as they laugh and dance. The night goes on and the two continue flirting and having a good time together. John is very attracted to Alice, and he would like to “take it to the next level” with her, but Alice turns him down several times. John, despite her constant rejection, grabs her strongly. Finally, they end up kissing and touching each other sexually.

### Appendix A.4. Vignette Four—Coercive Strategy: Physical Force; Narrative Framing: Passive Voice

John is out with some friends in a bar in the city. He has been watching a girl for a while that he really likes but, until now, he has not decided to approach her. She is dancing with some friends and seems to be interested in him because she has glanced at him several times. John decides to approach her, and he introduces himself. She responds by saying that her name is Alice, and the two begin to talk as they laugh and dance. The night goes on and the two continue flirting and having a good time together. John is very attracted to Alice, and he would like to “take it to the next level” with her, but Alice turns him down several times. John, despite her constant rejection, grabs her strongly. Finally, John ends up kissing her and touching her sexually.
